# Making fall prevention routine in primary care practice: perspectives of allied health professionals

**DOI:** 10.1186/s12913-018-3414-1

**Published:** 2018-08-03

**Authors:** Jeannine Liddle, Meryl Lovarini, Lindy Clemson, Lynette Mackenzie, Amy Tan, Sabrina W. Pit, Roslyn Poulos, Anne Tiedemann, Catherine Sherrington, Chris Roberts, Karen Willis

**Affiliations:** 1 Faculty of Health Sciences, The University of Sydney, Cumberland Campus C42, PO Box 170, Lidcombe, NSW 1825 Australia; 2 University Centre for Rural Health, Sydney Medical School, The University of Sydney, Sydney, NSW Australia; 3 School of Public Health & Community Medicine, University of New South Wales, Sydney, NSW Australia; 4 Musculoskeletal Health Sydney, School of Public Health, The University of Sydney, Sydney, NSW Australia; 5 Sydney Medical School – Northern, The University of Sydney, Sydney, NSW Australia; 6 Melbourne Health, La Trobe University, Parkville, VIC Australia

**Keywords:** Allied health, Implementation, Inter-professional collaboration, Qualitative methods

## Abstract

**Background:**

While there is strong evidence that fall prevention interventions can prevent falls in people aged 65 and over, translating evidence into routine practice is challenging. Research regarding how allied health professionals (AHPs) respond to this challenge is limited. As part of the Integrated Solutions for Sustainable Fall Prevention (iSOLVE) project, this study aimed to explore how AHPs were making fall prevention practice routine in primary care and the factors that influenced their fall prevention practice.

**Methods:**

In-depth qualitative interviews were conducted with fifteen AHPs who had attended evidence-based workshops associated with the iSOLVE project. AHPs had backgrounds in physiotherapy, occupational therapy, exercise physiology and podiatry. Interviews explored how fall prevention was being incorporated into routine practice and the factors that influenced routinisation, including the project workshops. Thematic analysis was used to analyse the data.

**Results:**

We found fall prevention was valued in practice and recognised as complex. AHPs worked through challenges relating to clients (multi-morbidity, complex living situations, client motivation), challenges working alongside other health professionals (understanding respective roles/overlapping roles, sense of competition, communication) and challenges associated with funding systems perceived as complicated and constantly changing. Despite these challenges, AHPs were adopting strategies for integrating fall prevention routinely. The iSOLVE workshops were perceived as important in supporting existing practice and in providing strategies to enhance practice.

**Conclusions:**

Policy makers, program managers, educators and AHPs can adopt strategies identified in this research for routinising fall prevention such as being alert that falls are common, asking every client about falls, having processes for assessing clients for fall risk, and having structured and evidence-based programs to work with clients on fall prevention. Adapting and streamlining funding systems are also important for facilitating fall prevention work.

## Background

Falls are a major health issue experienced by 1 in 3 people aged 65 and over every year. They are the major cause of injury in this age group [1], with an estimated 30% of falls requiring medical care and up to 1 in 5 falls leading to serious injury such as hip fracture [2]. Annual direct medical costs for fall-related injuries in the United States have been estimated at more than US$30 billion [3]. In Australia, more than half of injury-related hospital admissions among older people are due to falls [4] and total health care costs of fall-related injuries in New South Wales (population over 7 million) have been estimated at AUS$558.5 million per year [5]. Even non-injurious falls adversely affect people’s everyday function, social participation and independence [6]. Given the significant impact falls have on older people’s lives and on health care systems, preventing falls is crucial.

Multi-component fall prevention programs that treat underlying conditions that contribute to falls, and incorporate strength and balance exercises and home environment modification, have been found to lessen the risk of falling [7]. However, routinely applying effective fall prevention interventions in practice is challenging [8–10]. Multiple barriers to implementation have been cited, for example, insufficient time for health professionals to address fall prevention in the context of competing demands and a focus on diagnosis and treatment of specific diseases [11, 12]; fragmentation of services across settings and service providers with limited or differing understandings of respective roles [13, 14]; inadequate reimbursement for fall prevention work which is multifactorial and complex [11, 12, 15] as well as perceived lack of interest and/or fatalistic attitudes towards falling by clients [13, 16, 17].

Allied health professionals (AHPs) have an important role in identifying and managing fall risk through, for example, exercise and physical therapy [18] and home assessment and modification [19]. Yet, empirical research to guide AHPs on how best to take up and sustain evidence based fall prevention interventions in routine practice is limited [20]. Research to date has focused on implementation challenges faced by health professionals in hospital, emergency department or clinic settings, or by general practitioners (GPs) or nurses in primary care settings [15, 21], rather than the specific challenges faced by AHPs such as occupational therapists or physiotherapists, especially those in private practice [13, 22]. In Australia, private practitioners are those either self-employed or employed in a small business and who receive direct payment for services from clients [23]. Other sources of income include reimbursement through private health insurers and Federal government rebates through the Department of Veterans Affairs and Medicare [24]. In contrast, public sector AHPs are employed directly in government (usually State) funded and operated services. Research in Australia involving private practice AHPs has examined the use of Enhanced Primary Care funding (a government funded program) as a mechanism for greater involvement of AHPs in chronic disease management where fall prevention interventions can be included [21, 25]. This research echoes the need for better care models and reimbursement systems for health professionals noted in other contexts. What is not known is how else to facilitate AHPs making fall prevention routine. Guidance for AHPs in primary care settings is needed to enable better integration of evidence-based fall prevention strategies with the realities of day-to-day practice.

Theory can provide insights into how health professionals implement and sustain changes in practice including making fall prevention routine. For example, the current study drew on the Normalization Process Theory (NPT) which proposes that new practices become the norm as a consequence of people working individually and collectively and is dependent on how people make sense of a new practice, develop skills in, engage with, enact and appraise the new practice [26]. NPT introduces four key concepts – *coherence*, where the new way of working makes sense to people who would be normalising the practice; *cognitive participation*, where people cognitively engage with the new way of working, thinking through how the work will happen; *collective action*, where people enact the new work in practice; and *reflexive monitoring,* where people appraise whether the new way of working has been worthwhile [26]. Further, in order for a practice to become normalised there must be institutional or policy support to do so, for example, support from key stakeholders in the organisation or appropriate reimbursement.

The current study was undertaken as part of the Integrated Solutions for Sustainable Fall Prevention (iSOLVE) project [27]. The project takes a whole of primary care approach supporting AHPs and GPs to routinise fall prevention in practice. As part of the project 238 AHPs attended interactive fall prevention training workshops staggered over 2015 and 2016. The workshops included the latest research evidence for fall prevention, discussion on how to implement evidence into practice and opportunity to be included in local referral lists used by GPs in the project. Separate workshops were held on exercise interventions, home safety, the LiFE program [28] and foot and ankle interventions [29]. Following workshop participation, we aimed to explore in this study, how AHPs were making fall prevention practice routine in primary care and the factors that influenced their fall prevention practice, including the project workshops.

## Methods

### Design

A qualitative approach using interviews was designed to explore the experiences of AHPs working in fall prevention. Interview studies elicit practitioners’ perceptions and experiences, and importantly enable incorporation of the context in which they work, as this is fundamental to understanding how practices are normalised. We undertook in-depth interviews and analysed the data thematically [30].

### Study participants and recruitment

We purposively invited AHPs working in primary care settings who had attended more than one workshop (*n* = 42). We sought to recruit AHPs from occupational therapy, physiotherapy, exercise physiology and podiatry and from public and private practice. Potential study participants were invited by email to a 30–60 min face-to-face or telephone interview.

### Data collection and analysis

Interviews took place during 2016 and 2017**.** Time from first workshop attendance to time of interview varied from 3 to 18 months. Most interviews were conducted by the first author (JL), who is an experienced qualitative researcher and interviewer. Two interviews were conducted by an allied health Honours Student supported by the research team. Depending on AHP’s preference, interviews were conducted face-to-face at the AHP’s workplace (6 AHPs) or by telephone (9 AHPs). An interview guide was used to explore participants’ current practice context and experience, how fall prevention fitted into their everyday work and the extent to which, and how, workshop information had been integrated into practice (see Table 1 for sample interview questions). Unscripted follow up questions allowed participants opportunity to clarify and elaborate on responses. The main points of the interview were summarised by the interviewer and fed back to the participant at the end of the interview, to allow final opportunity to comment before interview closure. Interviews were audio-recorded and transcribed verbatim by the interviewer. The interviewer (JL) took comprehensive notes during and immediately after one interview where the participant declined audio-recording due to privacy concerns.

**Table 1:** Sample interview questions

Example questions	
- Can you describe to me the current practice settings you work in?	
- How well does fall prevention fit into your everyday practice?	
- How do clients respond to the fall prevention work you do with them?	
- Having gone to the workshops, what, if anything, are you doing differently?	
- How have you worked with colleagues to implement changes in practice?	
- Can you tell me about anything you would have liked to have implemented from the workshops but you haven’t been able to?	
- Is there anything else you’d like to say about fall prevention, the workshops or the iSOLVE project before we finish the interview?	

Interview recordings were listened to several times as part of the transcription process and transcripts and interview notes were read multiple times. Two transcripts were independently coded by four members of the research team. Similarities and differences in coding were discussed, leading to an agreed code listing, which was applied to the remaining transcripts and interview notes. No new codes were identified from the final three transcripts, indicative of data saturation [31]. Analysis proceeded iteratively with constant comparison between the data and emerging themes by the use of memos, reflexive notes, concept mapping and discussion among research team members until major conceptual themes were agreed [32]. N-Vivo 11 was used to manage interview data and document the analysis [33].

## Results

### Study participants

Fifteen workshop participants (13 women, 2 men) were interviewed. Participants had backgrounds in physiotherapy (6), occupational therapy (OT) (4), exercise physiology (EP) (2) and podiatry (3). Ten participants had been in practice for more than 10 years. Twelve participants were working in the private sector, of whom five ran their own business, three were employees in a small business, and four were employees in larger private sector organisations, for example, a not-for-profit aged-care organisation and a private rehabilitation hospital. Three AHPs were employed in either clinical, education and/or management roles in public sector organisations, coordinating and/or providing fall prevention programs and services in the community.

### Overview of major themes

**Fig. 1:**
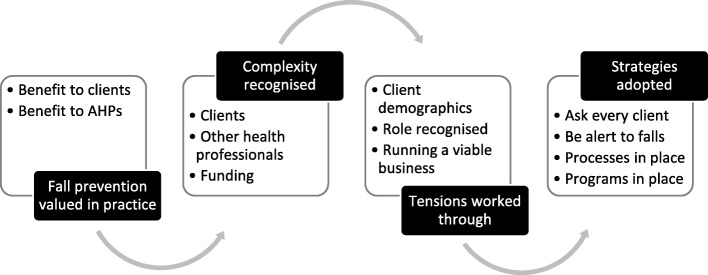
Major themes – Process of AHPs integrating fall prevention into routine practice

In normalising fall prevention work, four major themes were evident. Figure 1 represents these themes as stages in a process. In the first stage (Theme 1), AHPs valued fall prevention in practice recognising benefits for themselves and their clients. In the second stage (Theme 2), AHPs recognised the complexity of fall prevention work including working with clients who had multi-morbidity, complicated living situations and varying motivation. Complexity was also evident in working with other health professionals, where roles were unclear or overlapping; where there was a sense of competition; or where communication between health professionals was limited. A constantly changing funding environment was an added complexity. In the third stage (Theme 3), AHPs worked through these various tensions, mindful of their client demographics and the realities of running a business or meeting organisational requirements. In the fourth stage (Theme 4), strategies were adopted for integrating fall prevention into routine practice. We conclude the findings with a brief overview of participants’ perceptions on the influence of the iSOLVE workshops on fall prevention practice (Theme 5). In reporting findings, individual disciplines for private sector participants are identified. Due to the small number of public sector AHPs, individual disciplines are not identified when reporting findings specific for these participants.

### Theme 1: AHPs valued fall prevention in practice

Fundamental to the routinisation of fall prevention in practice was AHPs believing in the value of fall prevention. Regardless of private or public practice setting or disciplinary background, participants valuing of fall prevention came about through seeing evidence of benefit to clients:

“... he hasn’t fallen since. He walks two hours a day, goes down to the shops … he does everything. He gets out all the time.” (Public AHP, ID1).

“... you give them a lot of skills to increase their functional independence, increase their confidence, reduce their anxiety and independently manage their falls risk factors.” (Private OT, ID9).

For Private OT, ID9, the sense of making a difference to clients contributed to professional interest and satisfaction - “The kind of work that I was doing within the falls team ... I really like. I find it really empowering.” Seeing the benefits for clients as well as to themselves reinforced the importance of fall prevention as part of everyday practice.

### Theme 2: AHPs recognised the complexity of fall prevention work

While clients were at the centre of AHPs practice, clients also posed challenges to fall prevention work. AHPs could see the interconnected nature of falls and recognised the “complexity of what falling does … and that involves physical, mental, their support systems, confidence. It’s everything” (Private podiatrist, ID15). Clients at particular risk of falls had multiple morbidities that meant AHPs treating one problem could instead create another problem. ID15 described a client with diabetic neuropathy, foot drop and shuffling, “so now I’ve chucked these whopping big Otoform®s under her toes, so that’s obviously going to cause a little bit of an imbalance issue...” AHPs had to gain the trust of clients and work with them to adapt clients’ living spaces. In going to a client’s home, AHPs could see potential fall hazards first hand. However, engaging with clients to make changes and ameliorate fall risk, added further complexity to fall prevention work. Clients did not necessarily see they had a particular risk of falls, or that hazards needed to be addressed, or that exercise would be beneficial. Several AHPs expressed the view that persuading clients to act was the most difficult part of their fall prevention work:

“I did a home visit with a gentleman who was 94 and he had never had a fall ... I had a lot of trouble even convincing him that a home visit might be a good idea.” (Private OT, ID12).

“So the tricky bit in physio is getting people to do it...if you’re talking to someone who’s never exercised in their life and trying to persuade them why to follow something - that is the hardest bit I think.” (Private physiotherapist, ID7).

The multifactorial nature of falls meant that different health professionals could contribute to different aspects of fall prevention with individual clients. However, working alongside other health professionals in delivering services added further complexity. Part of this challenge was around role clarity – AHPs knowing their own role and the role of others, a challenge compounded when there was overlap in skills and experience across disciplines, for example, physiotherapists, EPs and OTs all had expertise in running exercise programs. This could contribute to a sense of competition and inter-professional rivalry between practitioners, especially where AHPs saw other health professionals as competing for business:

“Physios go in and take over everything because they’re dedicated to that sort of stuff and the patients trust them more with the exercises, so we don’t really get that.” (Private podiatrist, ID15).

Skepticism about the value of what other health professionals did in relation to fall prevention was expressed by AHPs in both private and public sector settings, however, recognition of how AHPs could complement each other’s services was also evident:

“...it’s mostly physios who send people through because they know that these people need to be motivated in another way and just giving them exercises is not enough. They need to get them to think through the issues ... so they send them to me, and then they get to ... consolidate what the physios been doing.” (Public AHP, ID1).

Furthermore, as noted by both private and public sector AHPs, communication between health professionals was limited, where AHPs could receive little or no information about clients referred to them and receive little or no feedback regarding clients they referred to other health professionals, compounding misunderstandings of how AHPs could work together.

The last element of complexity concerned a funding system which was perceived as complicated, constantly changing, had the potential to compromise continuity of care and was inadequate to meet demand. Private sector AHPs could be reimbursed for their fall prevention work through multiple publicly funded sources, private health insurers, or directly from clients. AHPs needed to know what funding options were available, how to access, and keep up to date with changing policies and funding opportunities:

“I still haven’t quite got my head around how it all works, the intricacies of all these new systems they have in place.” (Private physiotherapist, ID7).

Private practitioners expressed concern that a change in approach from government or private health insurers could mean their businesses getting “caught out” (Private physiotherapist, ID3). Two AHPs spoke of how they were funded for discrete services only, for example, assessment only or for a time limited period, and were then required to refer clients onto others for ongoing management, which could compromise continuity of care.

### Theme 3: Working through the tensions of integrating fall prevention into routine practice

Having recognised the multiple complexities involved, AHPs worked through the various tensions of routinising fall prevention in practice. Having a high proportion of clients aged 65 years and over with a greater risk of falls was a compelling reason for AHPs integrating fall prevention into routine practice regardless of private or public setting:

“... really anyone over a certain age if you’ve been unwell is actually falls prevention ... no matter what the original issue was.” (Private physiotherapist, ID7).

“... so you’re talking about that elderly population...it’s quite an everyday occurrence – falls prevention.” (Public AHP, ID8).

For public sector AHPs interviewed, fall prevention was recognised by their employing organisations as part of their role. For private sector AHPs, the proportion of time spent on fall prevention work varied. For those employed in larger private organisations, fall prevention represented “a very high percentage of my work” (Private OT, ID12), with increasing client demand for fall prevention services being a catalyst, in some cases, for their employment:

“... I was actually brought on ... because before that we only had two people in the team and they were finding it a bit hard to cope with the increasing volume of people wanting balance interventions.” (Private physiotherapist, ID10).

While most AHPs in private practice did not specifically tie their business to fall prevention and retained a generalist orientation, one private AHP had decided to specialise in fall prevention and balance, and market their services accordingly, believing in the value of a specialised practice. For some, a tension existed between providing more fall prevention services as part of their model of care and running a viable business:

“... the problem is that it’s crap for business ... so, although we come from the right place of caring, well I do – I come from the right place of caring, but you have to remember that you’re supposed to be making money for your time.” (Private podiatrist, ID15).

For other private sector AHPs, seeing the success of fall prevention strategies for improving clients’ lives had motivated them to incorporate balance improvement into every client’s program and use client word of mouth about their programs to grow the business and generate ongoing revenue:

“That’s what we focus on, client success, and the business grows from there … we’ve got to generate business through getting great results with our clients and getting them to refer.” (Private EP, ID4).

### Theme 4: Adopting strategies for integrating fall prevention into routine practice

AHPs adopted various strategies to make fall prevention routine including: asking every client about falls, being alert to falls as a common issue for their clients, having processes in place for assessing clients for falls risk and having structured programs in place to work with clients on fall prevention whether individually or in a group (see Table 2 for further details). Which strategies were used depended in part on the nature of each AHP’s practice, for example, Public AHP, ID1’s work focused on delivery of group-based programs, however, clients were still asked if they had experienced falls when they registered for the program. AHPs with a high proportion of older clients tended to ask every client about falls. Others with a wider age range of clients were alert to falls as a potential issue for their older clients. Some used standardised assessment forms to capture key information, however, Private physiotherapist, ID3 felt standardised forms “makes you close your eyes”. Rather, as an experienced clinician, it was important for ID3 to be open to observing what was happening with individual clients in their own environments and pick up issues not captured on the form.

**Table 2:** Strategies for integrating fall prevention into routine practice

Strategies	Example quotes
Ask every client about falls	“Every patient we consider it ... it’s a standard question we ask everyone whether they come in for neck pain, shoulder pain or if they’ve had a hip replacement, we ask everyone their falls history.” (Private physiotherapist, ID11)
Be alert to falls as a common issue relevant to many clients	“I’d say about half of them have been admitted because of a fall ... usually they’ve had an injury ... they’ve had a long hospital stay and they’re deconditioned and their mobility is reduced and they don’t have the confidence now ... so everyday I’m probably addressing falls in some kind of way in the community.” (Public AHP, ID8)
Have processes in place for assessing clients for risk of falls	“Some of them specifically come in for the [fall prevention] program, but others will come in with ... say a musculoskeletal impairment and then during assessment I will identify that there is also a balance component in it or a risk of falls because of other components that they’ve got.” (Private physiotherapist, ID10)
Have structured programs in place for working with clients on fall prevention	“… this is a simple exercise program ... we might not use every exercise with every patient but we’re aiming for them to do the whole program.” (Private podiatrist, ID5)

Many AHPs interviewed were using the strategies described as part of their own practice rather than influencing what other health professionals were doing. However, some self-employed business owners were in positions to make changes across a practice which affected what other health professionals did in regard to fall prevention, including making decisions on offering group and/or individualised services and programs and providing fall prevention services in clients’ homes:

“I think it’s up to us as a business and individual podiatrists that we employ, to say ‘I need to see you for a falls prevention assessment. Come back’.” (Private podiatrist, ID5).

In addition, some self-employed AHPs as well as public sector AHPs acted as fall prevention educators within their local networks.

### Theme 5: Perceptions of the influence of the iSOLVE workshops on practice

From the perspective of both private and public sector AHPs, and across the disciplines interviewed, fall prevention workshops were important in supporting existing practice and/or in providing strategies to enhance practice (see Table 3 for further details). Many were reassured that their practice was in line with “the right stuff” (Private physiotherapist, ID7) and were encouraged to use ideas and techniques from the workshops in their practice, for example, Private physiotherapist, ID3 reported more testing of balance with clients’ eyes closed and on unstable surfaces. Private OT, ID12 was “much more aware of asking about details”. Private OT, ID9 used the assessment tools discussed at the workshops when the organisation was updating its own client assessment processes. Others described refocusing their practice in line with research evidence.

**Table 3:** Perceptions of the influence of the iSOLVE workshops on practice

Example quotes	
“… having been to the workshops I’m much more likely now to say, right we’re going to really look at the circumstances of this fall and look at what really caused it and look at how we can prevent it.” (Private OT, ID12)	
“... [I’m] making sure there’s as much dynamic balance exercises as possible and incorporating it more into everyday life, using little strategies that we went through, like turning when the kettle’s boiling, standing on one leg, or doing some side stepping exercises, little things like that, trying to get people to change habits.” (Public AHP, ID8)	
“What I found really helpful was some of that research about how it’s balance exercise and lower limb strengthening exercises that shows an improvement in balance and reduction in falls ... that’s made me focus more on that, because that’s where the research is, so that’s where my practice needs to be as well.” (Private EP, ID2)	
“It’s just a matter of getting the program up and running and ... slot it in as an appointment type ... and that’s when we can integrate the falls prevention program ... we don’t have the foot exerciser, but we are using the marbles, foot movements and the Thera-Band®... and I’m printing out a list of things for the patient to do, giving them the link to [the] video. We’ve now got two CDs that we can lend to people, so we are actually doing it, which is good.” (Private podiatrist, ID5)	

The workshop on foot and ankle interventions detailed a comprehensive program which was new to many workshop participants. Consequently, AHPs described being more aware of foot issues in relation to fall prevention; were contemplating how to reinforce that in their practice and made sure clients took their shoes off. Private podiatrist, ID5 was incorporating that whole program into routine practice and actively engaging with other AHPs and GPs about the service, noting “they refer me a lot of work for falls prevention, but only since they’ve known I’ve been to the seminar and that I’ve started to talk about it.” Despite the positive feedback indicated above, some AHPs felt the workshops had not addressed the critical issue of motivating clients and saw that as a continuing gap in their training.

## Discussion

AHPs were alert to falls as a common issue with their clients. While acknowledging the challenges associated with the complexity of fall prevention work, they described taking steps to routinely incorporate fall prevention into everyday practice. Some asked every client about falls. Many had processes in place for assessing clients for falls risk and some were using specific fall prevention programs. Having an underlying belief in the importance of fall prevention to their practice was an important motivating factor for doing fall prevention work routinely. AHPs noted the difficulty of motivating some clients to make changes that would lessen fall risk and suggested behavioural change strategies as an area for future professional development. Other studies have observed that while older people acknowledge fall prevention as important, many do not consider fall prevention as personally relevant, linking falls to physical incapacity, advanced age and dependency [34, 35]. Given the multifactorial nature of falls and the complexity of preventing falls in the context of clients’ individual lives, fall prevention work needs to draw on knowledge and skills from multiple health disciplines [36]. AHPs found working with other health professionals complex especially when roles overlapped or were unclear. The workshops had value in improving inter-professional understanding and collaboration and supporting practice by providing opportunities for networking across disciplines; by reassuring AHPs they were taking an evidence based approach; by providing resources that AHPs were now using; and by stimulating AHPs to think about and implement additional strategies for implementation.

Governments increasingly recognise that existing disciplinary silos in health care systems need new care models that involve greater inter-professional collaboration [37]. However, and consistent with previous studies, our study still noted limited communication between service providers as a barrier to both inter-professional working and continuity of care [11, 13] as well as issues around funding [11, 12, 15]. Importantly, in our study, AHPs emphasised complexity and changing funding models for fall prevention as issues, in addition to inadequate funding. While a tension existed for some private practice AHPs between doing fall prevention as part of their model of care and running a viable business, others had seen opportunities to develop their business based on positive client word-of-mouth and by promoting their fall prevention expertise and services within their local networks. Other researchers have noted the ethical dilemma faced by health professionals in private practice to balance their desire to provide high quality services with the realities of running a successful business [38, 39]. More in-depth research could better identify which factors allow AHPs to successfully build viable businesses around routine falls prevention with the potential to translate those success factors to other businesses.

The workshops presented evidence supporting fall prevention practice around exercise, home safety and foot and ankle interventions. From a theoretical perspective, our major themes indicated normalisation of some of these practices was occurring, consistent with that espoused in Normalization Process Theory [26]. Consistent with the NPT concept of *coherence,* AHPs understood what fall prevention encompassed, why preventing falls was important and what the potential benefits were to both themselves and their clients. Benefits of an evidence based approach made sense and was consistent with practice goals and professional desire to help clients. Having attended workshops, AHPs thought about how they might adapt current practices to incorporate workshop learnings. Some AHPs were reassured their current practices were consistent with workshop content. However, fully integrating fall prevention in practice was complex. Some were grappling with multiple stakeholders and funding mechanisms and expressed doubt in their own ability to motivate clients or the wisdom of building their business model on prevention. Other AHPs felt confident in their ability to work through the complexity and were willing to further invest their resources in setting up additional programs at their practices and were in the process of thinking through how that would happen. This process of thinking through how normalisation could occur and in some cases, committing to moving forward with the new way of working was consistent with the NPT concept of *cognitive participation* [40]. *Collective action* was evident where AHPs were taking charge on fall prevention within their practice and sphere of influence. Some were already enacting elements of the iSOLVE approach that they found easy to adapt to current work practice, for example, using additional assessment tools or purchasing and using additional equipment. Some AHPs who were business owners or were in positions of influence, were engaging with their work teams (through team meetings, educational sessions) and enacting new programs at the practice. Some AHPs were actively engaging with health professionals outside the practice to promote their services and expertise in fall prevention. However, in the reality of everyday practice, some areas of evidence based practice were seen as needing a longer time to organise or would not be put in place as the barriers to make routine outweighed the benefits. The interview process itself represented an opportunity for AHPs to reflect on the impact of the workshops on routine practice as they identified ways to evaluate whether changes in practice had been worthwhile (*reflexive monitoring*), for example, through feedback from clients, direct observation and measurement of client progress and feedback from other health professionals.

The study had several limitations. A small number of workshop participants volunteered to be interviewed, and these may have had a particular interest in fall prevention. As participants in the current study were also participating in longitudinal surveys as part of the larger iSOLVE project, we decided that formal member checking here was not necessary and would overburden participants [41]. However, we did at the time of interview summarise the main points, allowing participants opportunity to comment before interview closure. The study was based in metropolitan Sydney and did not focus on issues specific to rural or disadvantaged communities. The time frame between attending workshops and being interviewed was short so sustainability of change beyond 18 months was not assessed. However, lessons can still be learnt from early adopters to translate routine falls prevention into practice. Our study included a range of AHP disciplines from a mix of small and large organisations and included twelve AHPs in the private sector who provided particular insight into the private sector context.

## Conclusion

Our study explored how AHPs were making fall prevention practice routine in primary care and the factors that influenced their fall prevention practice. Consistent with Normalization Process Theory, AHPs believed in the value of fall prevention work. Individually, and in some cases collectively, AHPs were appraising their current practice in line with what was recommended in the workshops, thinking through what more could be done or had begun to normalise some practices. In making fall prevention routine, AHPs were faced with many challenges such as motivating complex clients with multiple problems, working collaboratively across inter-professional boundaries, meeting organisational and professional goals, while at the same time, trying to make a living. Our examples, showing how some AHPs in real world practice have worked through these complexities, can assist other AHPs looking to incorporate fall prevention in their own context. Local, inter-professional workshops in specific fall prevention interventions was one area of support for AHPs, however, additional supports are needed for sustained implementation. Examples include AHPs being able to measure the benefits of fall prevention interventions and enhanced communication and collaboration among health professionals through local networks. Policy makers, program managers, educators and AHPs could use and promote strategies described by AHPs here. Additionally, adapting and streamlining funding systems in the Australian context would further assist routinising fall prevention in primary care practice beyond the iSOLVE project.

## Abbreviations

AHPs: Allied health professionals
EP: Exercise physiologist
GPs: General practitioners
ID: Participant identification number
iSOLVE: Integrated Solutions for Sustainable Fall Prevention
LiFE: Lifestyle Integrated Functional Exercise
NSW: New South Wales
OT: Occupational therapist

## References

[1] Centers for Disease Control and Prevention, National Center for Injury Prevention and Control. Web-based injury statistics query and reporting system (WISQARS) [online]. 2005. https://www.cdc.gov/injury/wisqars. Accessed 12 Feb 2018.

[2] Rubenstein LZ (2006). Falls in older people: epidemiology, risk factors and strategies for prevention. Age Ageing.

[3] Burns ER, Stevens JA, Lee R (2016). The direct costs of fatal and non-fatal falls among older adults – United States. J Saf Res.

[4] NSW Department of Health (2010). New South Wales falls prevention baseline survey: 2009 report.

[5] Watson W, Clapperton A, Mitchell R (2010). The incidence and cost of falls injury among older people in New South Wales 2006/07.

[6] Berry SD, Miller R (2008). Falls: epidemiology, pathophysiology, and relationship to fracture. Curr Osteoporos Rep.

[7] Gillespie LD, Robertson MC, Gillespie WJ, Sherrington C, Gates S, Clemson LM, Lamb SE (2012). Interventions for preventing falls in older people living in the community. Cochrane Database Syst Rev.

[8] Fixsen D, Scott V, Blasé K, Naoom S, Wagar L (2011). When evidence is not enough: the challenge of implementing fall prevention strategies. J Saf Res.

[9] Fortinsky RH, Baker D, Gottschalk M, King M, Trella P, Tinetti ME (2008). Extent of implementation of evidence-based fall prevention practices for older patients in home health care. JAGS.

[10] Goodwin V, Jones-Hughes T, Thompson-Coon J, Boddy K, Stein K (2011). Implementing the evidence for preventing falls among community-dwelling older people: a systematic review. J Saf Res.

[11] Baker DI, King MB, Fortinsky RH, Graff LG, Gottschalk M, Acampora D, Preston J, Brown CJ, Tinetti ME (2005). Dissemination of an evidence-based multicomponent fall risk-assessment and management strategy throughout a geographic area. JAGS..

[12] Tinetti ME, Gordon C, Sogolow E, Lapin P, Bradley EH (2006). Fall-risk evaluation and management: challenges in adopting geriatric care practices. The Gerontologist.

[13] Milisen K, Geeraerts A, Dejaeger E (2009). Use of a fall prevention practice guideline for community-dwelling older persons at risk for falling: a feasibility study. Gerontology.

[14] Turnbull C, Grimmer-Somers K, Kumar S, May E, Law D, Ashworth E (2009). Allied, scientific and complementary health professionals: a new model for Australian allied health. Aust Health Rev.

[15] Child S, Goodwin V, Garside R, Jones-Hughes T, Boddy K, Stein K (2012). Factors influencing the implementation of fall prevention programmes: a systematic review and synthesis of qualitative studies. Implement Sci.

[16] Horne M, Skelton D, Speed S, Todd C (2014). Falls prevention and the value of exercise: salient beliefs among south Asian and white British older adults. Clin Nurs Res.

[17] Yardley L, Bishop FL, Beyer N, Hauer K, Kempen GIJM, Piot-Ziegler C, Todd CJ, Cuttelod T, Horne M, Lanta K, Holt AR (2006). Older people’s views of fall-prevention interventions in six European countries. The Gerontologist.

[18] Michael YL, Whitlock EP, Lin JS, Fu R, O’Connor EA, Gold R (2010). Primary care-relevant interventions to prevent falling in older adults: a systematic evidence review for the U.S. Preventative Services Task Force. Ann Intern Med.

[19] Clemson L, Mackenzie L, Ballinger C, Close J, Cumming R (2008). Environmental interventions to prevent falls in community dwelling older people: a meta-analysis. J Aging Health.

[20] Lovarini M, Clemson LM, Dean C (2013). Sustainability of community-based fall prevention programs: a systematic review. J Saf Res.

[21] Grant A, Mackenzie L, Clemson L (2015). How do general practitioners engage with allied health practitioners to prevent falls in older people? An exploratory qualitative study. Australas J Ageing..

[22] Middlebrook S, Mackenzie L (2012). The enhanced primary care program and falls prevention: perceptions of private occupational therapists and physiotherapists. Australas J Ageing.

[23] Keane S, Lincoln M, Rolfe M, Smith T (2013). Retention of the rural allied health workforce in New South Wales: a comparison of public and private practitioners. BMC Health Serv Res.

[24] Merritt J, Perkins D, Boreland F (2013). Regional and remote occupational therapy: a preliminary exploration of private occupational therapy practice. Aust Occ Ther J.

[25] Foster MM, Cornwell PL, Fleming JM, Mitchell GK, Tweedy SM, Hart AL, Haines TP (2009). Better than nothing? Restrictions and realities of enhanced primary care for allied health practitioners. Aust J Prim Health..

[26] May C, Finch T (2009). Implementing, embedding and integrating practices: an outline of Normalization Process Theory. Sociology..

[27] Clemson L, Mackenzie L, Roberts C, Poulos R, Tan A, Lovarini M, Sherrington C, Simpson JM, Willis K, Lam M, Tiedemann A, Pond D, Peiris D, Hilmer S, Pit SW, Howard K, Lovitt L, White F (2017). Integrated solutions for sustainable fall prevention in primary care, the iSOLVE project: a type 2 hybrid effectiveness-implementation design. Implement Sci.

[28] Clemson L, Fiatarone Singh MA, Bundy A, Cumming RG, Manollaras K, O’Loughlin P, Black D (2012). Integration of balance and strength training into daily life activity to reduce rate of falls in older people (the LiFE study): randomized parallel trial. BMJ.

[29] Spink MJ, Menz HB, Fotoohabadi MR, Wee E, Landorf KB, Hill KD, Lord SR (2011). Effectiveness of a multifaceted podiatry intervention to prevent falls in community dwelling older people with disabling foot pain: randomised controlled trial. BMJ.

[30] Braun V, Clarke V (2006). Using thematic analysis in psychology. Qual Res Psychol.

[31] Francis JJ, Johnston M, Robertson C, Glidewell L, Entwistle V, Eccles MP, Grimshaw JM (2010). What is an adequate sample size? Operationalising data saturation for theory-based interview studies. Psychol Health.

[32] Corbin J, Strauss A (2008). Basics of Qualitative Research. Techniques and Procedures for Developing Grounded Theory. 3^rd^ ed.

[33] NVivo qualitative data analysis software (2015). QSR International Pty Ltd. Version 11.

[34] Yardley L, Donovan-Hall M, Francis K, Todd C (2006). Older people’s views of advice about falls prevention: a qualitative study. Health Educ Res.

[35] Hughes K, van Beurden E, Eakin EG, Barnett LM, Patterson E, Backhouse J, Jones S, Hauser D, Beard JR, Newman B (2008). Older persons’ perception of risk of falling: implications for fall-prevention campaigns. Am J Public Health.

[36] McKenzie G, Lasater K, Delander GE, Neal MB, Morgrove M, Eckstrom E (2017). Falls prevention education: interprofessional training to enhance collaborative practice. Gerontol Geriatr Educ.

[37] NSW Ministry of Health. Health Professionals Workforce Plan 2012-2022. Revised 2015. Sydney: NSW Ministry of Health; 2015.

[38] Davies JM, Edgar S, Debenham J (2016). A qualitative exploration of the factors influencing the job satisfaction and career development of physiotherapists in private practice. Man Ther.

[39] Millsteed J, Redmond J, Walker E (2017). Learning management by self-employed occupational therapists in private practice. Aust Occ Ther J..

[40] Murray E, Treweek S, Pope C, MacFarlane A, Ballini L, Dowrick C, Finch T, Kennedy A, Mair F, O’Donnell C, Ong BN, Rapley T, Rogers A, May C. Normalisation Process Theory: a framework for developing, evaluating and implementing complex interventions. BMC Med. 2010;8:63. 10.1186/1741-7015-8-63 PMC2978112 20961442

[41] Birt L, Scott S, Cavers D, Campbell C, Walter F (2016). Member checking: a tool to enhance trustworthiness or merely a nod to validation?. Qual Health Res.

